# How HIV-1 Uses the Metabolite Inositol Hexakisphosphate to Build Its Capsid

**DOI:** 10.3390/v17050689

**Published:** 2025-05-09

**Authors:** Leo C. James

**Affiliations:** MRC Laboratory of Molecular Biology, Francis Crick Avenue, Cambridge CB2 0QH, UK; lcj@mrc-lmb.cam.ac.uk

**Keywords:** HIV-1, IP6, retroviruses, capsid, structure, virus replication, virus assembly

## Abstract

The HIV-1 capsid is one of virology’s most iconic structures, yet how it assembles has long remained elusive. Remarkably, the capsid is made from just a single protein, CA, which forms a lattice of ~250 hexamers and exactly 12 pentamers. Conical capsids form inside budded virions during maturation, but early efforts to reproduce this in vitro resulted instead in open-ended tubes with a purely hexameric lattice. The missing component in capsid assembly was finally identified as the metabolite inositol hexakisphosphate (IP6). Simply mixing soluble CA protein with IP6 is sufficient to drive the spontaneous assembly of conical capsids with a similar size and shape to those inside of infectious virions. Equally important, IP6 stabilises capsids once formed, increasing their stability from minutes to hours. Indeed, such is the dependence of HIV-1 on IP6 that the virus actively packages it into virions during production. These discoveries have stimulated work from multiple labs into the role and importance of IP6 in HIV-1 replication, and is the subject of this review.

## 1. Introduction

The HIV-1 capsid is a protective container, reaction vessel, and delivery system for all stages between cell fusion and genome integration. There are many excellent reviews of these essential roles (e.g., [1]), and so this article will instead focus on how capsid is built and stabilised. Specifically, the role of the recently discovered assembly co-factor IP6 will be discussed in relation to both HIV-1 and other lentiviruses and its potential use by more diverse retroviruses. Nevertheless, for the purposes of understanding the capsid’s importance, a brief overview of its functional roles is provided here. The capsid contains the viral RNA and the enzymes—reverse transcriptase and integrase—necessary to copy the genome into DNA and integrate it into the host genome. By concentrating RNA and reverse transcriptase inside of an enclosed volume, the capsid acts as a reaction vessel and ensures that DNA synthesis is efficient [2,3]. Moreover, by sequestering the newly synthesised DNA inside of a protective container, the capsid prevents it from being sensed or destroyed by host immune proteins and enzymes [4,5]. The capsid is also a delivery system responsible for mediating transport to the nucleus on microtubules [6] and, once at the nuclear pore, entry into the nucleus itself [7,8,9]. Once inside of the nucleus, the capsid moves adjacent to regions of actively transcribing chromatin ready for uncoating and integration [7,10,11]. The exact spatiotemporal nature of uncoating and the manner in which it is triggered are subjects of current intense research by multiple groups [8,11,12,13,14].

## 2. Role of IP6 in Immature Lattice Formation and HIV-1 Production

**Figure 1:**
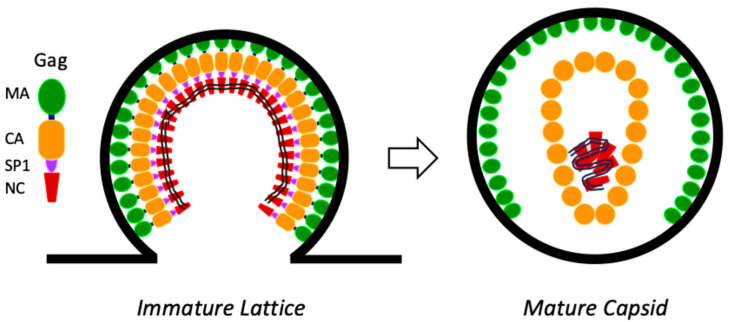
**HIV-1 capsid formation is a two-step process.** New HIV-1 virions assemble at the plasma membrane. The Gag polyprotein forms a hexameric immature lattice on the inside of the budding particle, anchored to the membrane via the matrix. Upon budding, the viral protease cleaves Gag to release CA, allowing it to form the mature capsid. Key: MA = matrix, CA = capsid, SP1 = spacer peptide 1, NC = NC capsid.

HIV-1 capsid formation is a two-step process (Figure 1). First, the polyprotein Gag assembles into an immature hexameric lattice during virion formation at the plasma membrane. Second, the viral protease cleaves Gag upon viral budding, liberating CA and allowing it to assemble into a mature capsid. Structural, biochemical, and cellular data suggest the involvement of IP6 in both of these steps.

**Figure 2:**
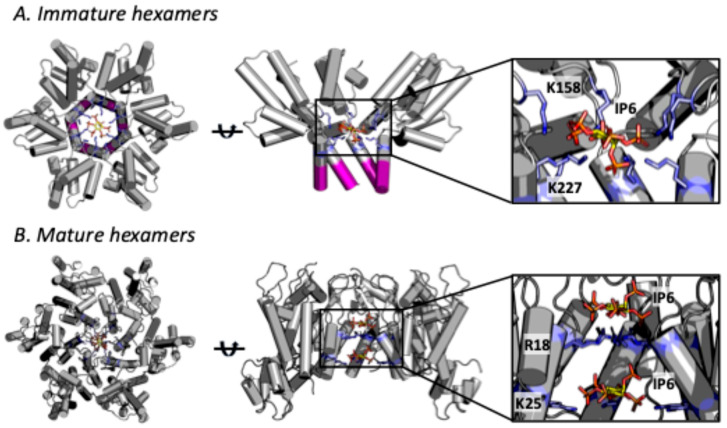
**Both immature hexamers and mature capsomers bind IP6.** (**A**) The immature lattice is made up of Gag hexamers that contain two rings of lysine residues—K158 and K227—located around a central channel (based on PDB 5I4T [15]). The two lysine rings (coloured blue) together coordinate a single IP6 molecule. Protease cleavage removes the SP1 region (coloured purple), disrupting the 6-helix bundle (6HB) and destabilising the lattice. (**B**) The mature capsid is made up of CA hexamers and 12 CA pentamers. Both hexamers and pentamers have two positively charged rings located around a central pore—R18 and K25 (coloured blue). R18 coordinates one IP6 molecule, whilst K25 coordinates a second IP6 molecule (based on PDB 6R8C [20]). Note that in the central images, only 4 copies within each hexamer are shown so that the IP6 binding site can be clearly seen. In all images IP6 is in standard atom colours.

IP6 is sufficient to induce the spontaneous assembly of immature virus-like particles (VLPs) from ‘CANC’ Gag protein comprising the CA (capsid) and NC (nucleocapsid) domains [15,16,17]. CryoET of in vitro assembled VLPs suggests that IP6 coordinates two rings of lysine residues at the centre of each immature lattice hexamer—K158 and K227 (CA numbering) [18] (Figure 2A). Complementary charged interactions between the 12 positively charged lysine side-chains and the six negatively-charged phosphates of IP6 is thought to stabilise hexamers and thus promote assembly of the immature lattice. Mutation of either lysine (K158 or K227) dramatically reduces IP6-driven VLP assembly in vitro [15]. Density consistent with an interaction between these lysines and IP6 can be seen in a co-crystal structure with Gag fragment CASP1 CA_CTD_–SP1 [15] and inside of hexamers in CryoET structures of immature virions [19]. The latter observation is consistent with data demonstrating that HIV-1 packages IP6 into virions as it buds from the cell [20]. This was demonstrated by producing HIV-1 in cells supplemented with tritiated inositol, allowing the number of IP6 molecules inside of each virion to be quantified [20]. Mutation of either lysine ring (K158 or K227) within immature hexamers reduces IP6 incorporation, demonstrating that they are responsible for actively enriching IP6 into the HIV-1 virion [20,21]. Thus, the process of immature lattice formation inside virions assembling at the plasma membrane is both driven by IP6 and ensures IP6 is packaged before budding from the cell.

HIV-1 must package IP6 because it is needed for capsid assembly and the metabolite is membrane impermeable. Indeed, each mammalian cell synthesises its own IP6 through the action of a series of biosynthetic kinases. The kinase IPMK is responsible for converting IP3 to IP4 and IP4 to IP5, while IPPK phosphorylates IP5 to IP6. The effect of knocking out (KO) these kinases on IP species from IP2 to IP5 has been accurately quantified [22]. IPPK KO leads to a 10-fold reduction in IP6 levels, whereas IPMK KO modestly reduces both IP5 and IP6. In addition to kinase knockout, the phosphatase MINPP1 can be over-expressed in cells to further reduce IP5 and IP6 levels to >100-fold lower than normal [22]. Experiments undertaken in these IP6-depleted cells have shown that IP6 is crucial for HIV-1 virion production [20,22]. The requirement for IP6 has been independently confirmed by multiple labs in both 293FT [23] and CD4+ T cell lines [24].

## 3. Role of IP6 in Mature Capsid Formation and HIV-1 Infectivity

The immature lattice disassembles when Gag is cleaved by the viral protease, liberating CA protein. The CA protein is then used to build the mature capsid, which is made up predominantly of hexamers and exactly 12 pentamers. The term ‘capsomers’ is used throughout this article to refer to hexamer and pentamer subunits collectively. Whilst there are only 12 pentamers, they are essential to allow a completely enclosed capsid structure to form. Their asymmetric distribution—seven at one end and five at the other—is also what gives the HIV-1 capsid its characteristic cone shape. Remarkably, IP6 alone is sufficient to induce the spontaneous assembly of mature capsids from CA protein [15]. IP6-driven capsid assembly occurs in vitro both rapidly and efficiently [25], but it requires 100-fold higher IP6 concentrations (~200 µM) than for immature VLP formation (~3 µM) [22]. IP6 is also essential for capsid formation inside virions, as evidenced by transmission electron microscopy data showing a lack of such structures inside particles budded from IP6-depleted cells [22].

Analogous to the immature lattice, there are two rings of positively charged residues at the centre of mature capsomers, and these are ideally positioned to engage with IP6—residues R18 and K25 (Figure 2B). Density consistent with IP6 can be seen in CryoET data of mature HIV-1 capsids from intact virions [26,27] and single-particle cryoEM of in vitro assembled capsid-like particles (CLPs) [28,29]. Crystal structures of HIV-1 capsid (CA) solved in the presence of IP6 also indicate binding [15,30]. Unlike with structures of the immature lattice, however, these data suggest that two IP6 molecules can bind per capsomer; one coordinated by R18 and a second by K25 [25]. In most cases, density for the R18-coordinated IP6 is stronger. There is also a difference between hexamers and pentamers, with stronger density for the second K25-bound IP6 in the latter [27]. A greater occupancy for the second IP6 in pentamers is intuitive, as the pore is necessarily smaller and the K25 side-chains closer together, increasing the strength of the interaction. Mutation of either R18 or K25 is sufficient to prevent the assembly of conical HIV-1 capsids in vitro. Mutating R18 abolishes assembly in the presence of IP6 but allows spherical particles to form in high salt conditions [25,31,32]. Mutating K25A allows assembly in either IP6 or salt, but, in both cases, only tubes are formed [25,32]. The smaller spherical particles seen with R18G necessarily have a higher proportion of pentamers, whilst K25A tubes are open-ended and comprise hexamers. These assembly data support the notion that IP6 coordination of K25 helps to build structures containing pentamers, whilst R18 engagement with IP6 promotes hexameric assemblies.

The importance of IP6 in mature capsid formation is also supported by CryoET data of mutant viruses. Significantly fewer conical capsids are seen in R18G and K25A virions, and these mutants are almost completely non-infectious [32]. Infection data support that of the two residues, R18 is the most critical. When passaged, K25A viruses regained infectivity by acquiring second site mutations that rescued conical capsid assembly without restoring the lost IP6 binding site [32]. These compensating mutations were non-charged substitutions located either within the pore or at interfaces within or between capsomers in the lattice. These results highlight that K25 and a second IP6 binding site are not necessary to form pentamers and a conical capsid.

## 4. IP6 Stabilises the HIV-1 Capsid

The role of IP6 in building the HIV-1 capsid is supported by both in vitro assembly data and the presence of the metabolite in assembled structures. However, IP6 likely continues to play a role even after the capsid has formed. This is because the capsid must remain intact for many hours after infection to allow the virus to reverse transcribe its RNA genome into DNA and traffic into the nucleus. Single molecule TIRF studies have shown that in the absence of IP6, the HIV-1 capsid is highly unstable and collapses within minutes, whereas in the presence of IP6, the capsid stays intact for hours [30]. The process of reverse transcription puts additional strain on the capsid. This is because DNA is far less flexible than RNA and increases pressure inside the capsid as it accumulates [33,34]. In vitro encapsidated reverse transcription (ERT) assays have shown that IP6 is critical to maintain capsid stability during DNA synthesis [30,35]. IP6 may also be important to maintain capsid integrity while allowing flexing of the lattice as the virus squeezes through the nuclear pore during nuclear entry [7,9,36]. This is because the IP6 binding site is located at the centre of capsomers, leaving inter-capsomer interfaces free to alter their twist and tilt angles. Variability in these angles is necessary to allow for the different curvatures implicit in a conical capsid, but it also allows for deformation in the lattice without breaking. Of course, breakage of the capsid is a functional inevitability, and IP6 may also play a role in altering capsid properties to allow for the completion of DNA synthesis to act as a mechanical cue for uncoating [37,38]. While the importance of IP6 in regulating capsid stability during post-entry infection is suggested by in vitro experiments, molecular dynamics (MD) simulations, and structural analysis, it is not supported by cellular data. The depletion of IP6 in target cells does not substantially reduce HIV-1 infectivity [20]. However, capsid mutants with decreased intrinsic stability (e.g., P38A) struggle to infect IPMK KO cells [39]. This important finding suggests that IP6 may be required post-entry but that wild-type capsids either maintain binding to the IP6 packaged during production or that their affinity to IP6 is sufficiently high such that they can scavenge the necessary molecules even when levels are reduced in kinase KO cells. Alternatively, WT capsids may be able to substitute IP6 for other polyanions in target cells (e.g., ATP or dNTPs).

## 5. HIV-1 Can Build Its Immature Lattice but Not Its Capsid Without IP6

Differentiating the role of IP6 in virion production versus virion infectivity has been crucial to understanding HIV-1’s dependence on IP6 during both immature lattice and capsid formation. This has revealed that IP6 plays an independent role at each stage [20]. Altering IP6 engagement with the mature capsid would be predicted to impact infectivity but not production because maturation occurs upon budding. Consistent with this, mutations R18G and K25A profoundly reduce virion infectivity but not production [32]. Conversely, altering IP6 engagement with the immature lattice would be predicted to impact viral production and this is indeed observed when removing either K158A or K227A from the immature lattice [21]. Somewhat surprisingly however, mutation of either lysine also results in a profound infectivity defect that is independent of the reduced production. For instance, K227I largely rescues production but has the same infectivity defect as K227A [20]. One way that altering the immature lattice can indirectly change infectivity is by influencing Gag processing. However, removing both charged rings (K158A/K227A or ‘KAKA’), which completely abolishes IP6 binding, has only a limited impact on viral production and Gag processing but reduces infectivity 10-fold [22]. These results highlight that engagement of the immature lattice with IP6 has separable and distinct implications for production and infectivity. This is further evidenced by the fact that more KAKA than wild-type virions are produced in IP6-depleted cells, even though the infectivity of the resulting virions is lower [22] This is consistent with the fact that the KAKA mutant, by lacking repulsive positive charges at the centre of immature hexamers, gains the ability to assemble VLPs in vitro without IP6 [22].

**Figure 3:**
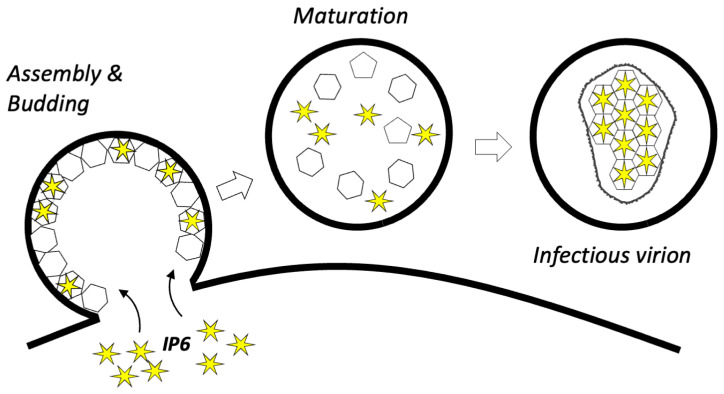
**HIV-1 uses its immature lattice to enrich IP6 into virions.** The immature lattice (shown as a stylized layer of hexagons) in the forming virion recruits IP6 (indicated as yellow stars) from the virus-producing cell. Upon budding, the immature lattice undergoes the process of maturation whereupon Gag is cleaved, releasing both CA protein and IP6. CA and IP6 can assemble within each particle, resulting in an infectious virion with a mature capsid made up of hexamers and pentamers.

The infectivity defect induced by mutations like K158A is partly explained because they disrupt IP6 recruitment into virions, and, without IP6, the mature capsid cannot then form. Quantification of IP6 levels inside of virions suggest that there are between 1 and 3 IP6 molecules for every mature capsomer [20,22]. Without IP6 enrichment, there are only enough molecules to stabilise 10–30% of capsomers, which is insufficient to allow capsids to form efficiently within virions [22]. Capsid formation by mutants like K158A is only restored with IP6 packaging, as demonstrated in forced evolution experiments. For instance, K158A infectivity is largely rescued through the acquisition of the second-site mutation T8I. T8I cannot directly impact capsid formation because it is removed during Gag proteolysis and thus not present in CA protein (Figure 2A). However, T8I stabilises the 6HB and thereby the immature hexamer, allowing it to rescue K158A by promoting IP6 engagement via the single remaining charged lysine ring provided by K227. Consistent with this model of rescuing capsid formation by restoring IP6 packaging, T8I is unable to rescue the KAKA mutant, which has no charged rings [22]. These findings also highlight that with only two mutations, Gag can efficiently assemble an immature lattice in the absence of IP6. This is a key point, as it suggests that the real reason HIV-1 maintains Gag binding to IP6 is not because it is an intrinsic requirement for immature lattice assembly but because the virus uses this as a mechanism to enrich IP6 into virions [22] (Figure 3).

Given the above, an obvious question is why HIV-1 has evolved to become dependent on IP6 in the first place. If it can mutate its immature lattice to assemble in the absence of IP6, why not do the same for the mature capsid? There are multiple possible reasons why IP6 binding is maintained by HIV-1. First, charged pores in the mature capsid may be essential to recruit nucleotides for encapsidated DNA synthesis [3]. IP6 may therefore be necessary to allow a capsid with such a strongly destabilising feature to be built in the first place. Second, IP6 may confer on the capsid a property of metastability, i.e., the ability to maintain a stable structure for many hours but one that can be rapidly disassembled upon IP6 dissociation. Third, IP6 may stabilise the capsid whilst maintaining plasticity—something that could be important for nuclear entry (as discussed above). Fourth, IP6 may be important for allowing CA to form both pentamers and hexamers and to set the equilibrium in favour of the latter—something that is essential in order to form a cone.

## 6. IP6 Usage by Other Lentiviruses

**Figure 4:**
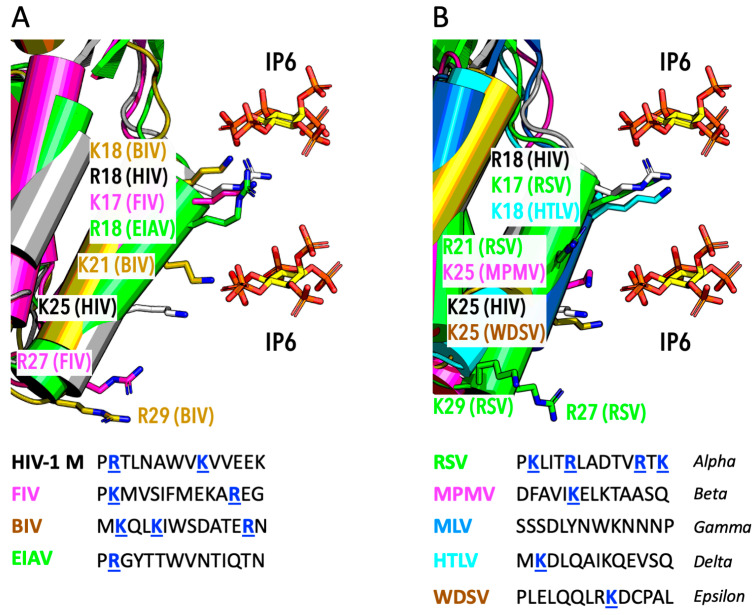
**Possible IP6-interacting residues in helix 1 of CA from diverse orthoretrovirinae.** (**A**) Alignment of N-term CA structures from a selection of lentviruses with HIV-1 (in grey). The position of two IP6 molecules, as found in HIV-1 around residues R18 and K25, is shown. (**B**) Alignment of N-term CA structures from a selection of diverse retroviruses with HIV-1 (in grey). (**A**,**B**) Sequences beneath the structural alignments highlight in blue the position of positively charged residues. Note that there are charged residues at multiple positions from the top to the bottom of helix 1 that face towards the IP6 binding site, which is at the centre of capsomers. All structures were created using AlphaFold3.

Another way of addressing the question of HIV-1’s dependence on IP6 is to consider whether IP6 is required by other viruses. The structural features that HIV-1 employs to bind IP6 are largely conserved across lentiviruses. All lentiviruses have two rings of lysine residues at the centre of their immature lattice hexamers. Binding to IP6 has not been systematically assessed, but there are CryoET data to support the co-ordination of IP6 by immature virions of HIV-2 [40] and in vitro assembled immature VLPs of HIV-2, SIV, FIV, BIV, and EIAV [15,41]. Lentiviruses also conserve a charged pore within mature capsomers. Most lentiviruses have R18, with a few, such as FIV and BIV, using a lysine at this position instead (Figure 4A). Lentiviruses also commonly have a second charged residue in helix 1. Primate lentiviruses conserve K25, two helix turns below R18, whilst others, like BIV, have a lysine at position 21, one helix turn below position 18. Some lentiviruses also have charged residues at the bottom of helix 1 that may be capable of binding IP6, such as R27 in FIV and R29 in BIV (Figure 4A). Whether IP6 promotes the in vitro assembly of mature CLPs from lentiviruses other than HIV-1 has yet to be shown. However, depletion of IP6 reduces the infectious viral titre of HIV-1 O, HIV-2, SIV, and FIV, consistent with their conservation of IP6 binding sites [20,23]. Thus, existing data suggest that IP6 may engage both the immature lattice and mature capsids of diverse lentiviruses.

## 7. IP6 Usage by Other Diverse Retroviruses

Lentiviruses are one of six genera in the orthoretrovirinae subfamily of retroviruses, the others being alpha, beta, gamma, delta, and epsilon retroviruses. Use of IP6 beyond the lentiviral genera is less well-characterised, and the structural features that mediate IP6 binding in lentiviruses—namely, the charged residues at the centre of immature hexamers and mature capsomers—are less well-conserved. There are structural data in support of IP6 binding to the immature lattice of only one virus outside of the lentiviral genera, the endogenous betaretrovirus HERV-K [42]. IP6 was not found in the immature structures of the extant betaretrovirus MLV [43], the alpharetrovirus RSV [44], or the deltaretrovirus HTLV-1 [45]. These findings are consistent with the fact that only HERV-K has lysine rings similar to those in HIV-1 immature hexamers. However, functional data on the potential use of IP6 during immature lattice assembly are limited. An in vitro immature VLP assembly study of Gag proteins from alpharetrovirus RSV, betaretrovirus MPMV, and gammaretrovirus MLV concluded that RSV VLPs could be promoted by IP6 and MLV by myo-inositol, but neither metabolite had any affect on MPMV [46]. However, a different study showed that RSV assembled VLPs independently of IP6 [44], although fewer RSV virions were produced in IPPK KO cells. Reduced infectious particle production in IPPK-KO cells has also been shown for MLV, although this was modest (three-fold), and the authors concluded that this was not a direct effect [23]. A more recent study reported no difference in MLV particle production or virion infectivity [47]. Taken together, the existing data is unclear whether diverse retroviruses use IP6 to build their immature lattice or use their lattice to package IP6 into virions.

There is more compelling evidence that IP6 may be used by diverse retroviruses to build and stabilise their mature capsids. Charged residues are more conserved at the centre of orthoretrovirinae capsomers (Figure 4B). Those retroviruses with a structurally equivalent positively charged residue to HIV-1 R18 include the alpharetrovirus RSV (K17) and the deltaretrovirus HTLV-1 (K18). The betaretrovirus MPMV, the gammaretrovirus MLV, and the epsilonretrovirus WDSV do not have an equivalent positively charged residue. None of these retroviruses conserve a structurally equivalent charged residue to HIV-1 K25, except, possibly, epsilonretrovirus WDSV. While betaretrovirus MPMV does not have an R18 or K25 equivalent, it does have a lysine residue one turn down the helix from R18 (equivalent to K21 in BIV) that could potentially allow for IP6 binding (Figure 4). RSV also has a charged residue at this position, R21, and two further basic residues at the bottom of helix 1, R27 and K29 (similar to R27 and R29 in FIV and BIV, respectively). MLV has no charged residues in helix 1; however, it does have an arginine (R3) in the β-hairpin, which forms the top of the pore in mature capsomers. In summary, structural analysis of diverse retroviruses suggests that many have charged residues in helix 1 located at a position that could allow for IP6 binding. Position 18 is the most conserved location on helix 1 for a basic residue amongst different lentiviruses and retroviruses more generally. However, basic residues can be found at every turn down the length of helix 1, suggesting that the exact position of IP6 within the capsomer pore could vary between viruses. Indeed, in a CryoET structure of RSV CASPNC CLP assembled in vitro in the presence of 100 µM IP6, there is density consistent with one metabolite molecule bound in an upright position between K17 and R21 [44]. However, the presence of a charged residue in helix 1 does not guarantee IP6 binding. Crystal structures of HTLV-1 CANTD solved in the presence of IP6 did not detect compelling evidence of IP6 binding despite the presence of K18 [48]. It may also be possible that IP6 binds elsewhere other than helix 1. Weak density was observed near R3 in a CryoET structure of mature MLV virions, which the authors speculated could indicate the presence of a negatively charged ion [43].

There has been limited investigation into the functional conservation of IP6 binding to diverse retroviral capsids. IP6 has been shown to promote the assembly of mature RSV capsids, and IP6 depletion in producer cells reduces the infectious titre of RSV [44]. It is unclear from these experiments at what stage RSV requires IP6 however, because production was not differentiated from particle infectivity. It is possible that reducing IP6 levels in producer cells results in virions with insufficient IP6 to drive proper RSV capsid maturation, explaining the poor infectious titre. A recent study on MLV found that IP6 depletion in target cells, via IPPK or IPMK knockout, reduced infection two- to three-fold [47], which would support IP6 interaction with the mature capsid. Consistent with this target cell effect, IP6 promoted endogenous reverse transcription and the recovery of intact cores from permeabilised virions [47], suggesting that IP6 may stabilise the capsid both prior to and during DNA synthesis, as shown for HIV-1 [20,30]. As MLV has no charged residues in helix 1 to coordinate IP6, the authors suggest that residue R3 is responsible for IP6 binding. However, IP6 depletion in producer or target cells does not phenocopy mutant R3A, which is non-infectious, suggesting that this residue is important for other reasons. In summary, current data hint that IP6 may be important in the replicative cycle of diverse retroviruses, but through interactions that are currently poorly understood.

## 8. Conclusions

Research within the last few years has transformed our understanding of how HIV-1 builds its capsid. We now know that the metabolite IP6 is the missing ingredient required for the assembly of new HIV-1 virions and the construction of a mature capsid. Many important questions remain however. Why has HIV-1 evolved to become dependent on this cofactor? What properties does IP6 confer on the capsid, and is this related to its ability to pass through the nuclear pore? How widespread is IP6 dependence amongst other retroviruses? Does differing IP6 engagement alter the functional behaviour of diverse retroviruses, and does this help explain their distinct pathologies and pandemicity? The answer to these questions awaits further research.

## Data Availability

No new data were created or analyzed in this study.
